# Japanese Traditional Herbal Medicine (Kampo Medicine) Improved Functional Hypothalamic Amenorrhea With Inherited Antithrombin Deficiency

**DOI:** 10.7759/cureus.83237

**Published:** 2025-04-30

**Authors:** Mitsuyuki Takamura, Yukari Nose, Yasuyuki Ota, Mayu Omori

**Affiliations:** 1 Center for Kampo Medicine, Mie University Hospital, Tsu, JPN

**Keywords:** hypothalamic amenorrhea, inherited antithrombin deficiency, japanese traditional herbal medicine, kampo medicine, venous thromboembolism

## Abstract

We report a case of a 28-year-old Japanese woman diagnosed with functional hypothalamic amenorrhea (FHA). Although oral estrogen-progestin therapy was initially recommended, laboratory tests revealed an inherited antithrombin (AT) deficiency, which is associated with an increased risk of venous thromboembolism (VTE). To mitigate this risk, we selected Kampo medicine (Japanese traditional herbal medicine) as an alternative treatment for amenorrhea. She was prescribed *Keishibukuryogan* and *Shigyakusan*, which led to the resumption of menstruation shortly after initiation. Subsequently, *Tokakujokito* was added to address persistent constipation, resulting in the regularization of her menstrual cycle and resolution of associated symptoms without adverse effects. This case suggests that Kampo medicine may offer a viable therapeutic alternative for managing menstrual disorders in patients predisposed to thrombosis.

## Introduction

Inherited antithrombin (AT) deficiency is an autosomal dominant thrombotic disorder, and affected individuals have a significantly higher risk of thromboembolism compared to healthy individuals [1-4]. In such patients, preventive anticoagulation therapy may be required prior to medical or surgical interventions that increase the risk of thrombosis.

However, common treatments for amenorrhea, such as estrogen replacement therapy, are also associated with an elevated thrombotic risk [5,6]. Several studies have concluded that estrogen-progestin combination therapies should be avoided or not strongly recommended in patients with AT deficiency due to the increased risk of venous thromboembolism (VTE) [5,7]. Women with AT deficiency also experience menstrual disorders, which occur across all age groups. Yet, as inherited AT deficiency is rare, there is a lack of evidence regarding effective treatments for menstrual disorders in this population. Therefore, treatment must be carefully selected on a case-by-case basis. For instance, in cases of functional hypothalamic amenorrhea (FHA) caused by estrogen deficiency, avoiding therapies that increase VTE risk is essential, but early intervention remains crucial to prevent long-term complications such as reduced bone mineral density [8]. This highlights the challenge of balancing risks and benefits in treatment selection.

As an alternative to estrogen-based therapies, Kampo medicine, a form of Japanese traditional herbal medicine, has been widely and historically used in Japan and is covered under the national health insurance system. Several Kampo formulations are approved for menstrual disorders and have already been utilized in clinical practice [9]. Additionally, Kampo therapy aims to improve a patient’s overall condition, regardless of the underlying disease. Herein, we report a case of a patient with FHA and inherited AT deficiency who was successfully treated with Kampo medicine.

## Case presentation

A 28-year-old Japanese woman presented to our department with complaints of amenorrhea. Six years prior, she had experienced significant psychological stress following a postgraduate entrance examination, leading to a 10 kg weight loss (from 47 kg to 37 kg; height: 156 cm), after which amenorrhea developed. She also reported long-standing vertigo, nausea, and primary focal hyperhidrosis. Three years earlier, she had been seen by a gynecologist who diagnosed her with FHA and initially recommended oral estrogen-progestin therapy. However, subsequent laboratory tests and a family history investigation revealed an inherited AT deficiency, leading to discontinuation of the therapy due to the heightened thrombotic risk. Although her body weight gradually returned to normal, amenorrhea, vertigo, and nausea relapsed after cessation of treatment. The patient was prescribed *Kamishoyosan* and *Tokishakuyakusan* by the gynecologist, but her symptoms did not improve.

She later visited our clinic seeking further Kampo therapy. At that time, she also complained of persistent severe constipation. She had no other significant medical history. Her social history included occasional alcohol use, and she was a non-smoker. A family history of AT deficiency was present in her maternal grandfather, mother, and younger sister. Her AT deficiency was identified as type I (quantitative deficiency), as confirmed by a hematologist at our institution. Ultrasonography showed no evidence of VTE. On physical examination, her height was 156 cm and weight was 47 kg (BMI: 19.3), with no fluctuation in weight over the past two years. Her blood pressure was 115/80 mmHg, and her pulse was regular at 70 beats per minute. Her complexion and skin were normal, and no abnormalities were found on chest auscultation. Tongue inspection revealed dark spots on a reddish background, suggesting the presence of blood stasis, according to Kampo diagnostics. Abdominal examination showed moderate tone, para-umbilical resistance and tenderness, hypochondriac resistance, and a brisk supra-umbilical pulsation, also indicative of blood stasis (a pattern mainly referring to microcirculation disorders) and liver qi stagnation (a pattern characterized by hindered qi movement) in Kampo terms. Blood and urine analyses were normal, except for low AT levels and relatively low estradiol (AT: 37.1%, soluble fibrin: 0.50 µg/ml, D-dimer: 0.04 µg/ml, estradiol: 24 pg/ml, follicle-stimulating hormone: 4.22 mIU/ml, luteinizing hormone: 3.45 mIU/ml).

**Figure 1:**
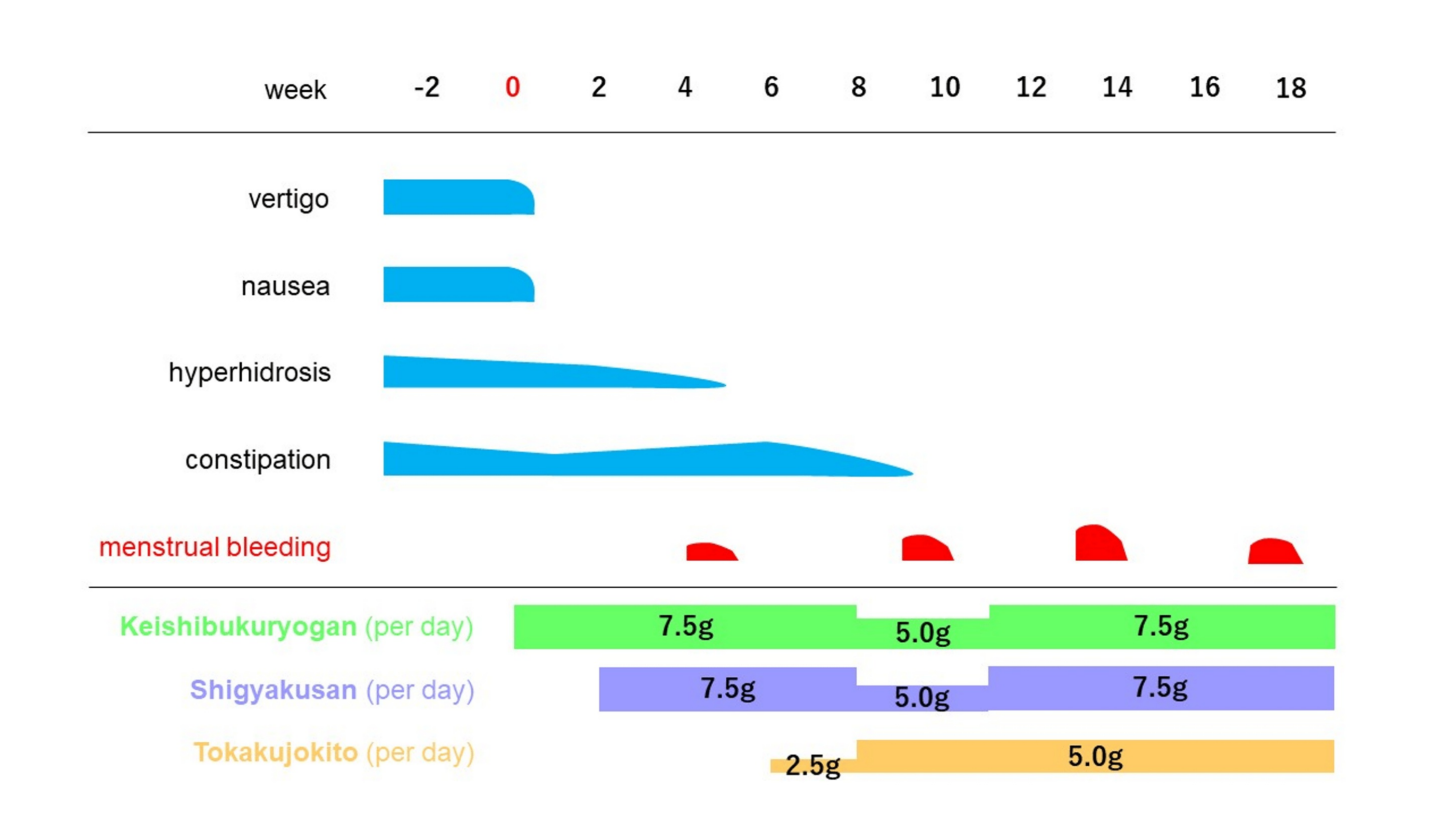
Clinical course.

Based on Kampo diagnosis, she was classified as having blood stasis, liver depression, and qi stagnation. We prescribed 7.5 g/day of *Keishibukuryogan* (Tsumura Co., Tokyo, Japan) for two weeks to address blood stasis, which promptly improved her vertigo and nausea. On her second visit, palmar hyperhidrosis persisted; therefore, we added 7.5 g/day of *Shigyakusan* (Tsumura Co., Tokyo, Japan) to treat liver depression and qi stagnation. Hyperhidrosis improved, and menstruation resumed shortly after administration of both formulas. To address her constipation, we added 2.5-5.0 g/day of *Tokakujokito* (Tsumura Co., Tokyo, Japan). Adjustments in dosage of the three formulas led to normalization of her menstrual cycle and volume, resolution of other symptoms, and maintenance of regular menstruation without adverse events (Figure 1). The patient was not taking any other medications during the treatment period.

After the menstrual cycle was regulated, treatment incorporating adjustments with other Kampo formulas was maintained for several years. Throughout this time, menstruation remained regular, and no adverse events were observed. Subsequently, the patient married and conceived naturally.

## Discussion

Antithrombin is a serine protease inhibitor (serpin) synthesized in the liver, which inactivates thrombin (factor IIa), factor Xa, and, to a lesser extent, other coagulation factors [1,2]. AT deficiency increases the risk of thrombosis in response to minor triggers such as pregnancy, the puerperium, trauma, or oral contraceptive use [1,5]. AT deficiency may be acquired (e.g., liver disease, protein loss, or increased consumption in disseminated intravascular coagulation) or inherited [1].

Inherited AT deficiency was first described by Egeberg in 1965 as an autosomal dominant condition [1]. It shows no gender, racial, or ethnic predilection. The estimated prevalence in the general population is 0.02% to 0.25%[2,10-12]. There are two main types: type I (quantitative), characterized by decreased levels of both antigen and activity, and type II (qualitative), with normal antigen levels but reduced functional activity [1]. Our patient was diagnosed with type I deficiency. A Japanese study reported that type I carriers have a significantly higher incidence of thrombosis compared to those with type II deficiency [2].

As in this case, estrogen-progestin therapy for menstrual control carries a significant thrombotic risk, prompting the hematologist to advise against its use. Although most data are derived from studies on postmenopausal women, one case-control study found that estrogen-progestin hormone replacement therapy was associated with a more than two-fold increased risk of deep vein thrombosis (DVT) [6]. The standard treatment for FHA typically includes estrogen-based therapy. Cognitive behavioral therapy (CBT) is also effective, particularly in stress-related cases, as it promotes the resumption of ovarian activity [13,14]. However, CBT remains less accessible in Japan due to a shortage of specialists.

Kampo medicine may help address these limitations. The concept of blood stasis (oketsu) in Kampo encompasses a wide range of pathological conditions, including menstrual disorders. It involves circulatory disturbances such as congestion in arteries, veins, and capillaries, including erythrocyte stagnation [15]. *Keishibukuryogan* is a representative anti-oketsu formula, composed of five crude drugs (Table 1), and is widely used in gynecology. Tsumura’s *Keishibukuryogan* is indicated for conditions such as inflammation of the uterus and its adnexa, endometritis, menstrual irregularities, and dysmenorrhea, etc. [9].

**Table 1:** List of compositions of Kampo medicines in the present study. Formulations of medicines referenced from [9].

Formula	Composition (JP: The Japanese Pharmacopoeia)
Keishibukuryogan	JP Cinnamon Bark 3.0 g, JP Peony Root 3.0 g, JP Peach Kernel 3.0 g, JP Poria Sclerotium 3.0 g, JP Moutan Bark 3.0 g
Shigyakusan	JP Bupleurum Root 5.0 g, JP Peony Root 4.0 g, JP Immature Orange 2.0 g, JP Glycyrrhiza 1.5 g
Tokakujokito	JP Peach Kernel 5.0 g, JP Cinnamon Bark 4.0 g, JP Rhubarb 3.0 g, JP Glycyrrhiza 1.5 g, Anhydrous Mirabilitum 0.9 g

*Keishibukuryogan* is generally considered more effective than *Kamishoyosan* or *Tokishakuyakusan* for resolving blood stasis, which may explain its success in this case. The abdominal findings could also have been compatible with *Kamishoyosan*; however, the reason for its lack of efficacy remains unclear. From the perspective of Kampo medicine, it must be concluded that the formula prescribed by the gynecologist did not match the patient’s *Sho* (pattern in Kampo manner).

Considering the patient's medication history, we prioritized the use of *Keishibukuryogan* due to her moderate abdominal tension and its recognized inhibitory effect on erythrocyte aggregation. Additionally, the patient occasionally experienced diarrhea with laxative use. Depending on the clinical response, we also considered the possibility of combining or substituting it with *Tokakujokito*. *Tokakujokito*, another anti-oketsu formula, is typically used in patients with severe constipation and includes rhubarb and mirabilite, which have cathartic effects not present in *Keishibukuryogan* (Table 1). Fortunately, the patient did not experience diarrhea as a side effect following administration of *Tokakujokito*.

*Keishibukuryogan* has been shown to promote arteriolar vasodilation, enhance blood flow velocity, and alleviate erythrocyte congestion [15]. These properties suggest a potential antithrombotic effect, indicating that it may be safely used for thromboprophylaxis in patients with AT deficiency, who are at a latent risk of thrombotic events. Furthermore, *Keishibukuryogan* has been suggested to exert menstrual-regulating effects through an estrogen-independent mechanism [16,17]. Therefore, it has a potential advantage in cases where estrogen-based treatments may not be suitable.

*Shigyakusan* (Table 1) is believed to relieve stagnation of liver qi and vital energy in both traditional Chinese medicine (TCM) and Japanese Kampo theory. According to Ninomiya, one of the symptoms of liver qi stagnation is palmoplantar hyperhidrosis, which has been shown to improve with *Shigyakusan* [18]. Stress-induced sweating is mediated by stimulation of the sudorific center in the frontal cortex, resulting in autonomic hyperactivity and palmoplantar sweating. In this case, hyperhidrosis improved following administration of *Shigyakusan*. Rodent studies suggest that *Shigyakusan* also exerts anxiolytic-like effects [19]. Given that psychological stress is a major trigger for FHA, *Shigyakusan* may have served as an alternative to CBT in this patient.

These findings suggest that Kampo medicine may serve as an effective therapeutic option for FHA in patients at thrombotic risk. To our knowledge, this is the first report demonstrating the marked efficacy of Kampo therapy in a patient with FHA and inherited AT deficiency. However, this case involves the simultaneous administration of three traditional Kampo formulas, making it impossible to determine the individual contributions of each to symptom improvement and limiting the ability to assess reproducibility. From the perspective of traditional medicine, *Keishibukuryogan* and *Tokakujokito* may have influenced the microcirculatory dynamics, while *Shigyakusan* likely exerted effects on psychological state, collectively contributing to FHA in a synergistic manner. This remains a hypothesis.

## Conclusions

We report the first known case of successful Kampo treatment for FHA in a patient with inherited AT deficiency, highlighting its potential as a safe and effective alternative to estrogen-progestin therapy in individuals at high risk of thrombosis. Kampo prescriptions, including *Keishibukuryogan*, *Shigyakusan*, and *Tokakujokito*, improved not only menstrual abnormalities but also associated symptoms such as vertigo, nausea, hyperhidrosis, and constipation. This case highlights the potential of Kampo medicine, grounded in individualized diagnosis and pattern differentiation, as a viable therapeutic option when conventional hormone therapy is contraindicated due to thrombotic risk.

## References

[1] Patnaik MM, Moll S (2008). Inherited antithrombin deficiency: a review. Haemophilia.

[2] Sekiya A, Taniguchi F, Yamaguchi D (2017). Causative genetic mutations for antithrombin deficiency and their clinical background among Japanese patients. Int J Hematol.

[3] Mahmoodi BK, Brouwer JL, Ten Kate MK (2010). A prospective cohort study on the absolute risks of venous thromboembolism and predictive value of screening asymptomatic relatives of patients with hereditary deficiencies of protein S, protein C or antithrombin. J Thromb Haemost.

[4] Stevens SM, Woller SC, Bauer KA (2016). Guidance for the evaluation and treatment of hereditary and acquired thrombophilia. J Thromb Thrombolysis.

[5] Girolami A, Stevanato F, Lazzaro AR (1988). Bilateral ileofemoral thrombophlebitis after ten contraceptive pills in a 25-year-old woman with antithrombin III deficiency. Acta Haematol.

[6] Douketis JD, Julian JA, Kearon C (2005). Does the type of hormone replacement therapy influence the risk of deep vein thrombosis? A prospective case-control study. J Thromb Haemost.

[7] Găman AM, Găman GD (2014). Deficiency of antithrombin III (AT III) - case report and review of the literature. Curr Health Sci J.

[8] Gordon CM, Ackerman KE, Berga SL (2017). Functional hypothalamic amenorrhea: an Endocrine Society clinical practice guideline. J Clin Endocrinol Metab.

[9] (2025). Table of the links of Kampo product informations in Japanese Pharmacopoeia (JP) and/or package insert. http://mpdb.nibiohn.go.jp/kconsort/kconsort.html.

[10] Kumar R, Chan AK, Dawson JE, Forman-Kay JD, Kahr WH, Williams S (2014). Clinical presentation and molecular basis of congenital antithrombin deficiency in children: a cohort study. Br J Haematol.

[11] Meade TW, Dyer S, Howarth DJ, Imeson JD, Stirling Y (1990). Antithrombin III and procoagulant activity: sex differences and effects of the menopause. Br J Haematol.

[12] Tait RC, Walker ID, Perry DJ (1994). Prevalence of antithrombin deficiency in the healthy population. Br J Haematol.

[13] Sowińska-Przepiera E, Andrysiak-Mamos E, Jarząbek-Bielecka G (2015). Functional hypothalamic amenorrhoea — diagnostic challenges, monitoring, and treatment. Endokrynol Pol.

[14] Shufelt CL, Torbati T, Dutra E (2017). Hypothalamic amenorrhea and the long-term health consequences. Semin Reprod Med.

[15] Tomita T, Hirayama A, Matsui H, Aoyagi K (2017). Effect of Keishibukuryogan, a Japanese traditional Kampo prescription, on improvement of microcirculation and oketsu and induction of endothelial nitric oxide: a live imaging study. Evid Based Complement Alternat Med.

[16] Noguchi M, Ikarashi Y, Yuzurihara M (2003). Effects of the Japanese herbal medicine Keishi-bukuryo-gan and 17beta-estradiol on calcitonin gene-related peptide-induced elevation of skin temperature in ovariectomized rats. J Endocrinol.

[17] Wang Z, Kanda S, Shimono T, Enkh-Undraa D, Nishiyama T (2018). The in vitro estrogenic activity of the crude drugs found in Japanese herbal medicines prescribed for menopausal syndrome was enhanced by combining them. BMC Complement Altern Med.

[18] Ninomiya F (2008). Clinical evaluation of perspiration reducing effects of a Kampo formula, Shigyaku-San, on palmoplantar hidrosis. Evid Based Complement Alternat Med.

[19] Tanaka M, Satou T, Koike K (2013). Anxiolytic-like effect of Shigyakusan extract with low side effects in mice. J Nat Med.

